# Gastroduodenal Emphysema with Portal Venous Air due to Congenital Duodenal Web in a Child: A Case Report and Review of Literature

**DOI:** 10.1155/2020/9897208

**Published:** 2020-01-17

**Authors:** Mihiri Chami Wettasinghe, Shanthini Rosairo, Samantha Kiriwattuduwa, Nuwan Darshana Wickramasinghe

**Affiliations:** ^1^Department of Radiology, Teaching Hospital, Peradeniya 20400, Sri Lanka; ^2^Department of Radiology, Faculty of Medicine, University of Peradeniya, Peradeniya 20400, Sri Lanka; ^3^Surgical Unit, Sirimavo Bandaranaike Specialized Children's Hospital, Peradeniya 20400, Sri Lanka; ^4^Department of Community Medicine, Faculty of Medicine and Allied Sciences, Rajarata University of Sri Lanka, Saliyapura 50008, Sri Lanka

## Abstract

Congenital duodenal web causing proximal duodenal obstruction leading to gastroduodenal emphysema is a very rare presentation in infancy. Due to persistent  peristalsis against the duodenal membrane, there is progressive stretching of the duodenal web leading to windsock deformity. We describe a rare case of a child with gastroduodenal emphysema and portal venous air due to duodenal obstruction secondary to a duodenal web. An eighteen-month-old male child, who was under investigation for failure to thrive, presented with a history of persistent projectile vomiting and progressive abdominal distension for two days. The abdominal ultrasound scan revealed air within the portal vein and in the wall of the stomach. Plain X-ray abdomen confirmed the presence of gas in the gastric wall and in the proximal duodenal wall. Upper gastrointestinal contrast study revealed complete obstruction at the second part of the duodenum. The child underwent emergency laparotomy, which revealed a duodenal web as the cause of the duodenal obstruction. During the surgery, windsock deformity was noted. This case illustrates that although rare, proximal duodenal obstruction due to duodenal web may present in early childhood and that alarming imaging features such as gastric emphysema and portal venous air could be associated with benign conditions.

## 1. Introduction

Gastroduodenal emphysema is a very rare clinical presentation in infancy [1, 2]. Evidence suggests that gastroduodenal emphysema is associated with gastric outlet obstruction secondary to pyloric stenosis and with duodenal obstruction [1, 2]. Out of the reported cases of gastric emphysema in infants, only a few cases were associated with duodenal obstruction [3]. Depending on the degree of duodenal obstruction, the age of presentation of the related clinical symptoms varies [3]. However, usually, these patients present in the first few months after birth [3]. Late presentations are rare, which need a high degree of suspicion for the correct diagnosis [4].

Congenital duodenal web is a known cause of neonatal duodenal obstruction, and depending on the type of the web, the clinical presentation and the age of the presentation may vary [5]. Only a limited number of case reports have been published on congenital duodenal web leading to duodenal obstruction and presenting with gastroduodenal emphysema in infancy [1, 2, 6]. Windsock deformity is another uncommon association with duodenal web reported in the literature [5, 7].

Portal venous air is very rarely associated with gastroduodenal emphysema [8, 9]. Even though portal venous air is usually considered as an ominous imaging finding, it could also have benign aetiologies [8, 9].

We present a very rare case of an eighteen-month-old child who developed gastroduodenal emphysema and portal venous air secondary to proximal duodenal obstruction due to a congenital duodenal web.

## 2. Case Presentation

An eighteen-month-old male child, who was under investigation for failure to thrive, presented with a history of frequent vomiting for two days. He had been having episodes of similar symptoms for the past one year, which were self-limiting and usually settling within one day. During these episodes, initially, the vomitus contained clear fluid; however, later it turned into coffee ground colour. In addition, he developed progressive abdominal distension. On examination, he was afebrile and not drowsy; however, he was mildly dehydrated. The child was in discomfort due to abdominal distension.

Initial basic haematological investigations such as full blood count, C-reactive protein, serum electrolytes, and urine full report did not reveal any significant derangements.

### 2.1. Imaging Findings

Initially, the child was referred for an abdominal ultrasound scan. It revealed multiple echogenic foci in the portal vein suggestive of intravenous air. In addition, a small amount of free fluid was seen in the abdomen. The stomach was distended with echogenic foci within the gastric wall. Furthermore, distal small bowel loops were collapsed.

**Figure 1:**
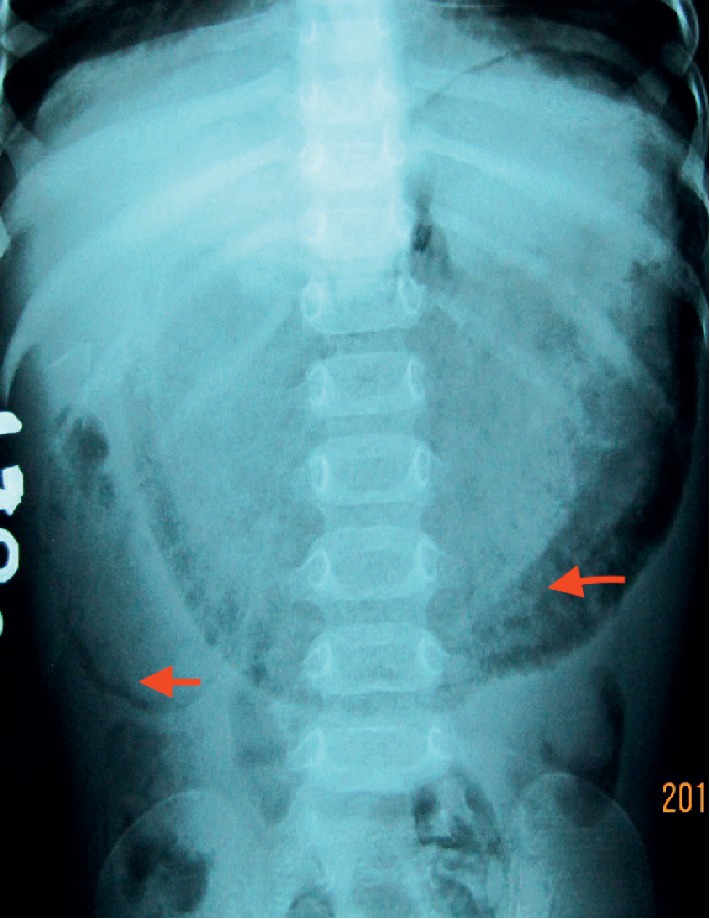
Plain X-ray abdomen showing gastroduodenal emphysema (red arrows).

A plain radiograph of the abdomen showed grossly distended stomach and proximal duodenum. Linear and circular air lucencies were present in the gastric wall and in the upper duodenum (Figure 1). The portal venous air was not seen in the plain radiograph.

The collective evidence from clinical, basic haematological investigations, plain radiography, and ultrasonography was suggestive of proximal duodenal obstruction. However, the reason for the gastroduodenal emphysema or the obstruction could not be explained by the available imaging findings, and it was decided to proceed with upper gastrointestinal contrast study in view of excluding gastric or midgut volvulus. Although the presence of portal venous air is considered as an ominous sign historically, as the foregut obstruction can cause portal venous air, an upper gastrointestinal contrast study was performed.

**Figure 2:**
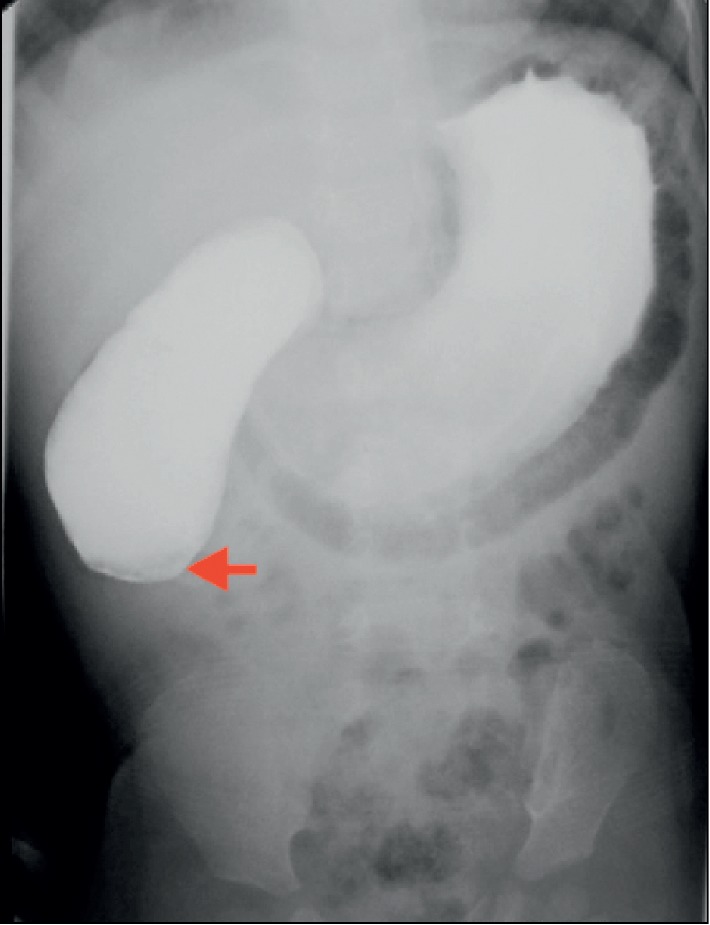
Upper gastrointestinal contrast study revealing complete obstruction at the 2nd part of the duodenum (red arrow).

The contrast study was carried out using low osmolar nonionic contrast media. The distended stomach and the proximal duodenum with gastroduodenal pneumatosis were seen during the fluoroscopic study. Although the stomach and the proximal duodenum were filled with contrast, no passage of contrast was seen beyond the obstruction (Figure 2). At the site of the obstruction, there was contrast accumulation with ballooning suggestive of a windsock deformity. However, a radiolucent line suggestive of a membrane was not noted.

The diagnosis of proximal duodenal obstruction was substantiated by the clinical and imaging findings. Following these investigations, the child was transferred to a specialized children hospital for the surgical intervention.

### 2.2. Surgical Findings

**Figure 3:**
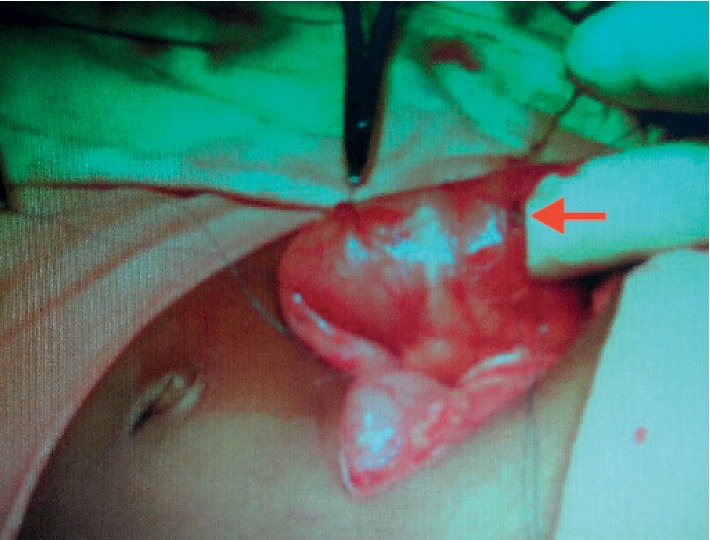
Surgeon passes the finger from the proximal duodenum. Vertical incision along the duodenum shows the web (red arrow).

During the surgery, the stomach and the proximal duodenum were found to be dilated and the crepitus of the stomach wall was felt. An incision was made on the proximal duodenum, and obstruction was felt at the second part of the duodenum. A thin membrane attached to the duodenal wall was revealed by a vertical incision made along the proximal second part of the duodenum (Figure 3).

**Figure 4:**
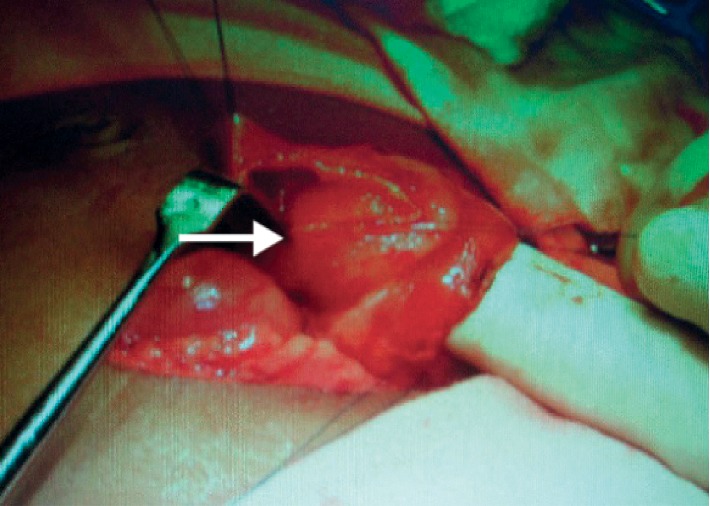
Obstruction at the 2nd part of the duodenum with windsock deformity (white arrow).

The web was stretched distally giving rise to a windsock deformity (Figure 4). The obstruction was proximal to the bile duct opening. The gastric contents were not bile mixed. The membrane was excised. The child had an uneventful recovery with no adverse or unanticipated events recorded postsurgically.

## 3. Discussion

The condition of gastroduodenal emphysema associated with duodenal obstruction secondary to congenial duodenal web seen in our patient is an exceedingly rare condition in early childhood [10, 11]. Comparison of the present case with reported cases of gastroduodenal emphysema due to congenial duodenal web is presented in Table 1.

Amongst the several postulated theories for the development of gastric emphysema in infancy secondary to obstruction, mucosal damage due to mechanical pressure developed in the distal obstruction is at the forefront. A tear in the mucosa is thought to be the cause for air tracking in the submucosa leading to emphysema [10, 15].

It is believed that following the mucosal damage, air tracking through tissue planes enter portal veins leading to portal venous air. Although portal venous air is historically considered to be an ominous sign, this had been described in more benign conditions like gastric outlet obstruction [8, 9]. Thus, a more conservative approach to patient management could be practiced rather than a radical surgical intervention, if the clinical and biochemical features are suggestive of a benign aetiology. Since the clinical and biochemical evidence did not suggest a grave aetiology for the gastroduodenal emphysema or portal venous air, but more of a gastric outlet obstruction in our patient, the contrast study was performed to determine the site and the possible cause of obstruction. This also avoided CT scanning, which would have exposed the child to a higher radiation dose.

In the literature, gastric emphysema is classified into two types, viz., emphysematous gastritis and gastric emphysema. It is important to differentiate these two entities as gastric emphysema has a benign self-limiting course, whereas emphysematous gastritis has more grievous course, which needs prompt intervention [8, 16, 17]. Radiologically, it is often difficult to differentiate the two entities and the differentiation is mainly based on the clinical presentation [1]. Literature suggests that the characteristic radiological finding in emphysematous gastritis is the presence of streaky and linear air in the gastric intramural area, whereas more round gas bubbles are seen in gastric emphysema [1, 17]. The emphysematous gastritis presents with acute abdomen and often with elevated inflammatory markers. In gastric emphysema, patients are often haemodynamically stable and do not demonstrate features of acute abdomen [8, 17]. Our patient was haemodynamically stable and had no features to suggest acute abdomen. Thus, careful evaluation by the clinical team helped in deciding the appropriate radiological investigations and final patient management.

Duodenal webs are small membranes, which consist of mucosa and submucosa and are usually seen in the second part of the duodenum. However, there are a few cases reported in the literature, in which the webs were found in the third and fourth part of the duodenum [7]. In this patient, the web was found in the second part of the duodenum. These are usually attached to the duodenal wall partially, though circumferential attachment is seen in few instances. In our patient, the web was attached circumferentially in the duodenum.

Usually, there is a small central pinhole aperture in the congenital duodenal membrane. Long-term pressure of peristalsis against the membrane leads to distal stretching of the web, forming an intraluminal pseudodiverticulum, which is known as a windsock diverticulum or windsock deformity [7].

Duodenal webs can cause complete or partial obstruction depending on the size of the central aperture. This affects the age of presentation and also the mode of the presentation in these patients. Obstruction of the aperture in the membrane with food particles or gradual onset atony with ineffective peristalsis against dilated proximal duodenum can result in delayed presentation [5]. These webs are seen as a thin radiolucent line in upper gastrointestinal contrast studies [10, 18]. In addition, the contrast pooling at the site of obstruction may give rise to typical windsock appearance on contrast studies [18]. In our patient, the windsock deformity of the web was noted in the contrast study as well as during the surgery.

The evidence suggests that there is an association between Down's syndrome and anatomical abnormalities in the duodenum [4, 6]. Most of the reported cases on paediatric gastric emphysema with duodenal obstruction have described the condition in children with Down's syndrome [1, 2, 10, 13]. However, our patient had no chromosomal abnormalities.

Gastric decompression and the treatment for the aetiology of the obstruction are known to relieve the gastric emphysema [1], and this was seen in our patient. Although this patient had normal serum electrolytes, these children usually present with hyponatraemia. Thus, before embarking on any surgical interventions, patient stabilization should be a priority.

Majority of the patients with duodenal webs present early during the first year of life, but atypical presentations as in our patient, should be anticipated. Even though rare, high degree of suspicion should be maintained when a child presents with features of proximal bowel obstruction.

## 4. Conclusions

This case clearly illustrates the importance of correlating clinical, biochemical, and imaging findings in formulating a final diagnosis and in patient management. Although presence of both the gastric emphysema and portal venous air are concerning, benign aetiologies can also cause these radiological findings and interpretation should be made considering clinical picture. Variable presentations of congenital duodenal webs should be borne in mind, as delayed diagnosis can lead to detrimental effects on the child.

## Figures and Tables

**Table 1:** Comparison of the present case with reported cases of gastroduodenal emphysema due to congenial duodenal web.

Case report	Age and sex	Clinical presentation	Clinical findings	Radiological findings	Surgical findings
Bahador and bagheri [12]	2 days, male	Bilious vomiting and failure to pass meconium	—	Plain X-ray: distended stomach pneumatosis of duodenal wall Upper GI contrast study: incomplete duodenal obstruction	Duodenal obstruction due to duodenal web

Alvarez et al. [6]	20 days, female	Nonbilious vomiting for 24 hours	Mildly dehydrated; abdominal examination: abdominal distension; biochemical investigations: moderate metabolic alkalosis	Plain X-ray: distended stomach and duodenum, gastric emphysema; USS abdomen: echogenic foci with reverberation in the gastric wall; upper GI contrast study: thin, partially obstructing web at the second part of the duodenum	Duodenal diaphragm with a central aperture

Kawano et al. [2]	7 months, male	Progressive vomiting and abdominal distension in a Down's syndrome baby	Abdominal examination: upper abdominal distension; biochemical investigations: normal	Plain X-ray: gastric pneumatosis, dilated duodenum; duodenography: membranous stenosis of the second part of the duodenum	Thick membrane with a pinhole in the second part of the duodenum, associated malrotation

Fernandes and smith [10]	8 months, female	Progressive nonbilious, nonprojectile milk regurgitation in a Down's syndrome baby	Abdominal examination: normal; biochemical investigations: metabolic alkalosis, hyponatraemia, hypokalaemia	Plain X-ray: gastric emphysema; CT abdomen: gastric emphysema, partial distal duodenal obstruction	Partial duodenal obstruction secondary to duodenal stenosis

Gupta [13]	9 months	Projectile vomiting in a Down's syndrome baby	Abdominal examination: distension of the epigastrium with visible peristalsis; biochemical investigations: normal	Plain X-ray: distension of the stomach with an unusual translucent “halo” around the stomach. Barium meal: distended stomach, gas in the stomach wall	Thick-walled muscular septum with a small opening in the centre

D'Cruz and Emil [1]	9 months, female	Progressive worsening of projectile vomiting for 5 days in a Down's syndrome baby	Lethargic and dehydrated; abdominal examination: normal; biochemical investigations: severe hypokalaemic, hypochloraemic, metabolic alkalosis	Plain X-ray: gastric emphysema; CT abdomen: partial duodenal obstruction, gastroduodenal emphysema	Partial duodenal obstruction secondary to a web in the proximal second portion of the duodenum

Thacker et al. [14]	10 months, female	Insidious onset of bilious vomiting for 2 days	Lethargic and dehydrated; biochemical investigations: normal	Plain X-ray: grossly distended stomach with intramural gas, pneumoperitoneum	Duodenal web at D4, severe diffuse gastritis with sloughing of mucosa and ulceration in intraoperative gastroscopy

Present case	18 months, male	Progressive, nonbilious vomiting and abdominal distension for 2 days	Mildly dehydrated; abdominal examination: abdominal distension; biochemical investigations: normal	USS abdomen: multiple echogenic foci in the portal vein; plain X-ray: grossly distended stomach and proximal duodenum with gastroduodenal emphysema; upper GI contrast study: gastroduodenal pneumatosis, windsock deformity	Duodenal web with windsock deformity
